# Imaging-Guided Surgical Decision-Making and Bone Healing in Mandibular Cystic and Tumor-like Lesions: A Case-Based Radiologic Observation

**DOI:** 10.3390/diagnostics16111677

**Published:** 2026-05-29

**Authors:** Ömer Uranbey, Filip Kulewicz, Özenç Beste Kandemir, Furkan Özbey, Büşra Ekinci, India Maag, Agata Małyszek, Maciej Janeczek, Maciej Dobrzyński, Kamil Nelke

**Affiliations:** 1 Department of Oral and Maxillofacial Surgery, Faculty of Dentistry, Aydın Adnan Menderes University, Aydın 09010, Türkiye; 2 Aesthetic Medicine and Dental Implantology Specialised Practice, Śniadeckich 53, 51-604 Wrocław, Poland; 3 Faculty of Dentistry, Oral and Maxillofacial Radiology, Afyonkarahisar Health Science University, Afyonkarahisar 03030, Türkiye; 4 Department of Medical Pathology, Faculty of Medicine, Aydın Adnan Menderes University, Aydın 09100, Türkiye; 5 Private Dental Office, 54-144 Wrocław, Poland; 6 Department of Biostructure and Life Animal Physiology, Wrocław University of Environmental and Life Sciences, Cypriana K. Norwida 31 Street, 50-375 Wrocław, Poland; 7 Department of Pediatric Dentistry and Preclinical Dentistry, Wrocław Medical University, Krakowska 26 Street, 50-425 Wrocław, Poland; 8 Maxillo-Facial Surgery Ward, EMC Hospital, Pilczycka 144, 54-144 Wrocław, Poland

**Keywords:** odontogenic cysts, marsupialization, preventive mandibular plating, cone-beam computed tomography, diagnostic imaging

## Abstract

**Background and Clinical Significance:** Mandibular cystic lesions are heterogeneous in biologic behavior, radiologic appearance, and surgical management. Treatment selection is influenced by lesion extent, cortical bone condition, structural stability, and proximity to adjacent teeth and vital anatomical structures. In large mandibular lesions, case-specific decision-making may range from staged decompression or marsupialization to single-stage enucleation, peripheral ostectomy, graft-assisted reconstruction, and preventive mandibular plating (PMP). **Case Presentation:** In the first case, a 60-year-old female presented with bilateral mandibular lesions: a dentigerous cyst on the right side, treated with marsupialization followed by enucleation, and a contralateral glandular odontogenic cyst (GOC) managed with primary enucleation alone. The second case involved a large, unilateral odontogenic keratocyst (OKC) managed with a radical approach, including enucleation followed by peripheral ostectomy and PMP. Histopathologic evaluation was performed in both cases to confirm diagnosis and support definitive treatment planning. Radiologic follow-up at 1 year demonstrated bone regeneration in all treated sites. **Conclusions:** This case report presents two different clinical cases involving three mandibular cystic lesions managed according to case-specific surgical indications and evaluated with standardized postoperative panoramic and CBCT imaging; limited supportive assessment of trabecular bone remodeling was also performed to further characterize radiologic healing patterns. The authors aimed to descriptively document postoperative bone condition, radiologic healing, and patient safety in these cases, and to highlight the most important surgical and radiological findings that may influence case-specific clinical decision-making.

## 1. Introduction

Odontogenic cysts of the jaws are typically slow-growing lesions that expand gradually, generating pressure on the surrounding bone and progressively weakening its structure. As they enlarge, they may compromise the vitality of adjacent teeth. When a cyst is associated with an impacted tooth, it may also lead to displacement of the tooth toward nearby anatomical spaces, including the maxillary sinus, nasal cavity, or mandibular ramus [1]. Clinically, these lesions often remain silent and are frequently discovered during routine dental examinations. If left untreated, and in some cases even after surgical intervention, these lesions may be associated with complications such as pathologic fracture, secondary or postoperative infection, and sensory disturbances related to inferior alveolar nerve involvement [2]. The likelihood of these outcomes increases with lesion size, underscoring the need for an appropriate treatment plan to achieve optimal clinical results.

Bone healing after surgery for mandibular cystic and tumor-like lesions is a highly case-dependent process influenced by both biologic and mechanical factors. Although histopathologic diagnosis remains essential, the choice of surgical approach is usually shaped preoperatively by radiologic findings such as lesion size, location, cortical thinning or perforation, deformation of the inferior mandibular border, relationship to impacted teeth and the inferior alveolar nerve, and the possible risk of pathological or iatrogenic fracture. For this reason, lesions with broadly similar radiolucent appearances may require markedly different treatment strategies, including simple enucleation, decompression or marsupialization, peripheral ostectomy, graft-assisted reconstruction, or preventive mandibular plating. As summarized in Figure 1, satisfactory bone healing may occur through several different clinical pathways, and no single reconstructive strategy is universally applicable to all mandibular defects. This concept is especially important in large lesions of the mandibular body, angle, and ramus, where surgical aggressiveness must be balanced against structural stability, neurosensory safety, and long-term bone regeneration.

Panoramic radiographs are widely used as the first-line imaging method for evaluating jaw cysts because they provide broad coverage and are readily accessible. However, their two-dimensional nature may limit the accurate assessment of lesion size, cortical bone thinning, and relationships with adjacent critical structures. Large, multilocular, or bilateral cysts can be particularly difficult to evaluate with panoramic imaging alone, as subtle cortical expansions or proximity to vital anatomical landmarks may be missed. Therefore, CBCT plays a central role in preoperative assessment by allowing a more accurate evaluation of lesion extent, cortical integrity, and the relationship to adjacent vital structures [3,4,5]. This additional radiologic detail may directly influence treatment planning, particularly in cases with extensive bone loss, cortical instability, or an increased risk of iatrogenic or pathological fracture during biopsy or surgery. To our knowledge, dentigerous cysts represent the most commonly encountered odontogenic cysts of the jaws after periapical (radicular) cysts. These lesions are characteristically associated with the crown of an unerupted tooth, most frequently the mandibular third molar. Smaller lesions often remain clinically silent. In contrast, large bilateral presentations in non-syndromic patients are exceptionally uncommon, with only a limited number of cases documented in the literature [5]. In our first case, a non-syndromic patient presented with such a rare bilateral manifestation, involving a large dentigerous cyst on one side and a contralateral glandular odontogenic cyst (GOC), further highlighting the clinical and pathological complexity of these lesions. There remains a need for more objective, quantitative methods to evaluate post-surgical bone regeneration beyond subjective radiological observation. Current literature often relies on qualitative descriptions, leaving a gap in standardized, imaging-based monitoring of bone stability and healing dynamics.

The present case report uses three different mandibular cystic lesions with distinct surgical pathways to illustrate how imaging findings may guide treatment selection and how postoperative radiologic follow-up may help interpret bone healing after treatment. Descriptive radiologic assessment was complemented by fractal analysis of trabecular bone remodeling to provide additional insight into healing patterns. Rather than presenting a pathology-matched comparison, this report is intended as an imaging and surgery-oriented observation of three different clinical scenarios in which treatment decisions were shaped by lesion extent, cortical bone condition, structural risk, and proximity to vital anatomical structures.

## 2. Case Description

### 2.1. Case 1

#### 2.1.1. Patient Information 

A systemically healthy 60-year-old female sought evaluation due to long-standing tooth loss (#36). Despite the duration, she remained asymptomatic, denying pain, swelling, or any other discomfort. Her medical and dental histories were unremarkable.

#### 2.1.2. Diagnostic Assessment

Intraoral examination (A) showed healthy mucosa without any evident pathological findings, such as vestibular expansion or fistula formation. Routine panoramic radiography (B) revealed well-circumscribed, corticated, unilocular radiolucent lesions involving both mandibular rami, with green and blue arrows delineating the cystic borders on the right and left sides, respectively (Figure 2). On the right side (green arrows), a larger lesion was associated with an impacted and inferiorly displaced third molar (#48) and extended superiorly toward the coronoid process, forming a sizable cystic cavity. On the left side (blue arrows), a smaller cystic lesion involved the impacted third molar (#38) and the ramus, without extension to the sigmoid notch.

In the present case, the identification of bilateral, well-demarcated cystic lesions associated with impacted mandibular third molars raised a strong radiologic suspicion of dentigerous cysts and prompted further diagnostic evaluation and treatment planning. The radiological differential diagnosis for well-demarcated pericoronal radiolucencies with cortical thinning included odontogenic keratocysts, unicystic ameloblastomas, glandular odontogenic cysts, and other odontogenic cystic or tumor-like lesions. Fibrous dysplasia and other fibro-osseous lesions may also be considered in the broader radiological differential diagnosis of mandibular expansile lesions; however, the absence of a typical ground-glass or mixed radiopaque/lucent pattern, ill-defined blending margins, and diffuse fibro-osseous expansion made fibrous dysplasia less likely in the present cases. Notably, this case is remarkable for remaining asymptomatic despite the considerable size of the lesions, without any mandibular fracture, jaw instability, occurrence of pathological fracture, or bone bending while eating.

For CBCT imaging, the Planmeca ProMax 3D Mid (Planmeca, Helsinki, Finland) device available at our hospital was utilized. The imaging parameters were as follows: field of view (FOV) of 16.0 × 6.2 cm, voxel size of 400 µm, image matrix of 400 × 400, and 155 slices. The exposure settings were 90 kVp and 8 mA. Coronal CBCT sections (A) allow mediolateral evaluation of the cystic lesion and adjacent cortical plates (blue arrows indicate the preoperative buccal and lingual cortical bone thickness in the right mandibular ramus region, while yellow arrows demonstrate the close proximity of the IAN to the teeth associated with the cystic lesions on both sides). CBCT images (B) show thinning of the lingual cortical plate bilaterally (arrows) and impacted third molars (#48 and #38). Axial CBCT image (C) demonstrates thinning of the buccal cortical bone (arrows) and the measured dimensions of the lesion. The CBCT examination revealed an intraosseous lesion on the right side measuring approximately 3.8 × 2 × 3.8 cm, extending from tooth #47 to the superior border of the ramus, with irregular margins and involvement of the mandibular canal. A similar but smaller lesion was observed on the left side, measuring approximately 3.2 × 1.5 × 3.2 cm, with the impacted tooth located in a more accessible position and the inferior alveolar nerve coursing lingually. On the representative CBCT sections shown in Figure 3, the thinnest residual buccal and lingual cortical bone measured approximately 0.67 and 0.70 mm on the right side, and 0.44 mm and 0.67 mm on the left side, respectively; the shortest measurable distance between the lesion/tooth complex and the inferior alveolar canal was approximately 0.50 mm on the right and 2.01 mm on the left. Red dots illustrate the course of the IAN in the mandibular region on sagittal CBCT views (D), reconstructed from sequential cross-sectional images. The patient-specific transparent 3D model (Biotechnica Engineering Co., Ltd., İstanbul, Türkiye) shows the wire (E), the inferior alveolar nerve (indicated by orange arrows), and the areas of cortical perforation (Figure 3). These radiographic findings showed cortical thinning, canal involvement, lesion extent, and mandibular ramus involvement. The lesions were associated with substantial bone loss. Postoperative healing, structural stability, and radiologic bone regeneration were documented during follow-up.

#### 2.1.3. Treatment Planning

In the surgical management of mandibular cysts, treatment planning depends on the lesion’s size, location, and relationship with surrounding structures. Most cysts can usually be managed safely through enucleation and extraction of the associated tooth. Large cysts, particularly those linked to deeply impacted third molars, carry a higher risk of pathologic fracture and nerve injury if enucleated directly. In such cases, marsupialization is often performed as a first-stage treatment.

#### 2.1.4. Surgical Procedure

In the present case, the right-sided lesion was extensive, associated with a deeply impacted third molar, and accompanied by marked cortical thinning and close canal relationship, which were considered to increase the risk of mandibular fracture and nerve injury if managed by primary enucleation. Therefore, marsupialization was initially performed. Under general anesthesia, a mucoperiosteal envelope flap was elevated to gain access to the cystic cavity. Upon entry, histopathological sampling was performed as a critical diagnostic step. Following incisional biopsy, drains were positioned within the cavity and stabilized by suturing to the buccal and lingual gingiva. The intraoperative photograph of the right mandible immediately after the first surgery is presented in Figure 4A, showing the drains placed following marsupialization. This approach was used as the first stage of treatment, followed by later radiographic and clinical follow-up [6,7]. The postoperative control radiograph obtained after the first surgery is presented in Figure 4B, showing the marsupialized right mandibular cyst with the drain in situ and the treated left-sided lesion. The marsupialization phase extended over approximately six months. During this period, the patient underwent two weeks of intermaxillary fixation (IMF) to minimize fracture risk, and the cystic cavity was irrigated regularly through the drain to maintain patency and hygiene. In contrast, the lesion on the left side was smaller, and the associated impacted tooth was located in a more superior position. Therefore, primary enucleation combined with tooth extraction was performed on that side. As a result, two different surgical strategies were applied in the same patient, tailored to the size, extent, and anatomical relationships of each lesion. The control radiograph obtained 6 months later (Figure 4C), after extraction of the impacted tooth and enucleation of the left mandibular cyst, demonstrates bone healing and resolution of the lesion. Pre-marsupialization vertical position of the tooth is presented in Figure 4D (blue arrow), and boundaries of the cyst (green arrows) are shown on the right side. Six months after marsupialization, radiographic evaluation (Figure 4E) shows a marked reduction in cystic dimensions (green arrows) and vertical eruption of tooth #48 (white arrow), facilitating safer definitive surgical management (Figure 4).

Microscopic examination of the right-sided, larger mandibular odontogenic cyst reveals (A,B) a cystic lining composed of non-keratinized cuboidal epithelial cells arranged in two to three layers, resting on a fibrous connective tissue wall without evidence of active inflammation. The epithelial lining appears thin and uniform, lacking rete ridge formation or epithelial proliferation. The underlying connective tissue consists of dense collagenous stroma with sparse fibroblastic cellularity and no inflammatory infiltrate. No keratin production, satellite cysts, or epithelial budding are identified in the examined sections (hematoxylin and eosin [H&E], ×100 HPF). Sections from the left mandibular lesion demonstrate a markedly thickened, non-keratinized stratified squamous epithelial lining with variable thickness and multiple intraepithelial microcystic formations (black arrows) ((C); hematoxylin–eosin [H&E], ×100). Multifocal dense aggregates of mucous goblet cells are identified within the epithelial lining (black arrows) at low and intermediate magnifications ((D,E); H&E, ×100 and ×200, respectively). In addition, numerous clusters of mucous goblet cells are present within the cyst wall (black arrows) ((F); H&E, ×100). These histomorphologic features are characteristic of a glandular odontogenic cyst (Figure 5).

#### 2.1.5. Radiological Follow-Up and Clinical Outcome

In the present case, panoramic radiography and CBCT imaging obtained at the one-year postoperative follow-up demonstrated (A–C) substantial bone healing and remodeling in both mandibular regions. In the present report, bone healing was descriptively assessed by radiologic evidence of trabecular infill, cavity reduction, and cortical reconstitution; nerve recovery by the clinical course of postoperative sensory disturbance; and structural stability by the absence of fracture, maintenance of mandibular continuity, and preserved occlusal function during follow-up. Marsupialization of the right-sided lesion (B) resulted in progressive reduction of the cystic cavity with peripheral-to-central bone formation (green arrows indicate bone regeneration within the lesion, whereas the red arrow marks the entry and exit orifices of the marsupialization tube), and (C) enucleation of the left-sided lesion achieved near-complete osseous regeneration (green arrows highlight bone healing after direct enucleation while yellow arrows indicate bone formation within the enucleation site). No postoperative infection, recurrence, or neurological complications were observed (D,E), and mandibular function and occlusion remained stable at 3–6-month and one-year follow-up evaluations (Figure 6).

### 2.2. Case 2

#### 2.2.1. Patient Information

A patient was referred for consultation because of pain and swelling in the right mandibular basis and angle (Figure 7). Local dentist evaluation indicated a follicular cyst appearance because of a retained ectopic third lower molar. At this stage, the radiological differential diagnosis included dentigerous cyst, odontogenic keratocyst, unicystic ameloblastoma, and other cystic odontogenic tumors, particularly because cortical thinning, cortical perforation, inferior border deformation, and mandibular canal involvement may overlap among these entities.

#### 2.2.2. Diagnostic Assessment

Because some lingual cortical bone loss was present, along with thickening of the inferior border of the mandible, a typical punch biopsy revealed the presence of an OKC (odontogenic keratocyst). Because the mandibular canal containing the inferior alveolar nerve was displaced and embedded within the lesion, and extracortical spread extended from the lingual cortex toward the lingual nerve, decompression was considered to avoid extensive surgery and potential nerve injury (A–D). For CBCT imaging, the Planmeca ProMax 3D Mid (Planmeca, Helsinki, Finland) device available at the hospital was utilized. The imaging parameters were as follows: field of view (FOV) of 16.0 × 6.2 cm, voxel size of 400 µm, image matrix of 400 × 400, and 155 slices. The exposure settings were 90 kVp and 8 mA. Because the preoperative biopsy confirmed an odontogenic keratocyst, a lesion with recognized recurrence potential, peripheral ostectomy was considered as part of the definitive treatment strategy rather than basing the surgical approach solely on lesion size or location.

#### 2.2.3. Treatment Planning

The patient was unwilling to undergo prolonged surgery; therefore, a decision was made to pursue a more radical approach involving bur ostectomy following cyst enucleation. Potential benefits of this approach include a shorter treatment duration, reduced reliance on a prosthetic obturator, fewer postoperative follow-up visits and bone-cavity irrigation sessions after cyst marsupialization, and a lower risk of pathological mandibular fracture. A one-stage approach was used. Decision-making in each case depends on the clinical assessment, thorough discussion with the patient in which all surgical options are presented, the patient’s approval and willingness to undergo a selected procedure, and the surgeon’s judgment regarding the optimal approach, while more radical strategies generally confer lower recurrence rates [5,6,7,8].

#### 2.2.4. Surgical Procedure

The OKC, along with teeth 47 and 48, was removed after a typical full mucoperiosteal flap was elevated. With high-speed Lindeman burs, the mandibular retromolar trigone was exposed. The OKC was removed with bone curettes while all bone cavity walls underwent a high-speed drill bone ostectomy. In the inferior border of the mandible, special care was taken to preserve the inferior alveolar nerve (IAN), while, because of the cortical lingual plate loss, a subperiosteal elevation helped in identifying the lingual nerve (LN). Both nerves were shifted to a safer and more controlled position to perform a radical bone ostectomy and guarantee more adequate surgical margins, especially since the OKC tends to relapse in around 5–37% of cases, depending on the retrospective studies [9,10,11]. OKC treatment options might include a number of different approaches; however, the presence of ortho- or hyper-keratosis in the OKC histo-pathological specimen should affect the decision-making similarly to the measurement of bone condition status [10,11,12]. Because CBCT demonstrated extensive bone loss with inferior border deformation, although without overt fracture, a PMP was planned (2.0 rigid plate with 6 screws, Medartis, Basel, Switzerland). After ensuring radical surgery to all bone walls, 20 mL fresh-frozen bone (FFB—allogeneic bone) from the bone bank (RCKiK, Katowice, Poland) was mixed with a total of 15 mL of the patient’s blood drained intravenously. After the preparation of a “sticky bone” (allograft bone crushed and mixed with blood), wound suturing was done with a combination of Nylon/PDS sutures 3.0/4.0. No artificial membranes or meshes were used. The wound healed uneventfully, with no evidence of inflammation, fracture, or wound dehiscence. Loss of sensation in the IAN + LN was noted postoperatively and Nurovit 320 mg twice a day was prescribed (1 tablet consists of: 100 mg wit. B1, 200 mg wit. B6, 0.2 mg wit. B12; 100 tabl.; G.L. Pharma, Gerot-Lannah, Wien, Austria) along with Thionerv-600 per os 600 mg (alfa-lipoic acid, 600 mg, Solinea, Lublin, Poland) once a day for three months of therapy.

#### 2.2.5. Radiological Follow-Up and Clinical Outcome

During follow-up, inferior alveolar nerve function recovered within three months, whereas lingual nerve-related sensory disturbance improved only partially, with approximately 80% recovery and residual deficit still present at the latest follow-up. PMP was removed after one year, and follow-up imaging showed radiologic consolidation of the grafted defect without evidence of OKC recurrence (E–H). After two years, no OKC recurrence was noted in either clinical or radiological follow-up.

Figure 7 presents a single-step surgical approach to a cystic lesion to illustrate an alternative management pathway. To better illustrate the range of treatment philosophies, Case 1 (marsupialization with staged treatment) and Case 2 (a single-step approach involving enucleation, bone grafting, and placement of a PMP) are presented as distinct management examples with different healing courses and reconstructive implications.

To appreciate the rationale and potential advantages of prolonged decompression, it is useful to observe its healing course alongside that of a single-stage radical ostectomy combined with immediate bone grafting and PMP. One-stage surgery should consist of a more radical approach with curettage and/or enucleation with ostectomy and local surgical high-speed burr radicalization. On the other hand, when inferior border deformation, cortical instability, or extensive bone loss is present on imaging, prophylactic plating may be considered to improve stability and reduce the risk of immediate, early, or delayed pathological fracture. The scope of cortical bone loss might affect bone perforation, the necessity for additional bone grafts, and decreased bone stability. In the following case 2, a one-step surgery was used.

In line with the methodology of Kaygisiz and Karsli [12], a region-of-interest-based fractal analysis (FA) was performed on both panoramic and CT images and revealed a consistent increase in fractal dimension over a 1-year period, reflecting radiographic bone remodeling in both cases. ROIs were standardized to 30 × 30 pixels and were placed within the trabecular bone of the healing region at anatomically comparable locations on preoperative and postoperative images. ROI selection was guided by fixed local anatomic landmarks and performed on corresponding postoperative images at the same axial level or the closest reproducible panoramic region. Cortical borders, root structures, mandibular canal boundaries, fixation hardware, graft margins, and obvious radiographic artifacts were intentionally excluded from ROI placement. FA was conducted using White and Rudolph’s box-counting method implemented in ImageJ software (version 1.52v; National Institutes of Health, Bethesda, MD, USA). In this descriptive case-based observation, the combined (marsupialization plus enucleation) approach was associated with a gradual fractal dimension (FD) progression, whereas direct enucleation showed a sharper postoperative rise; however, these patterns should be interpreted descriptively and not as evidence of comparative treatment effect. (A) Preoperative panoramic radiograph (OPG) of Case 1 and (B) the corresponding 1-year postoperative panoramic radiograph of Case 1; (C) preoperative panoramic radiograph of Case 2 and (D) the corresponding 1-year postoperative panoramic radiograph of Case 2. (E) Preoperative axial CT slice of Case 1 and (F) the 1-year postoperative axial CT slice of Case 1 obtained at the same axial level; (G) preoperative axial CT slice of Case 2 and (H) the 1-year postoperative axial CT slice of Case 2 obtained at the same axial level (Figure 8).

**Figure 8:**
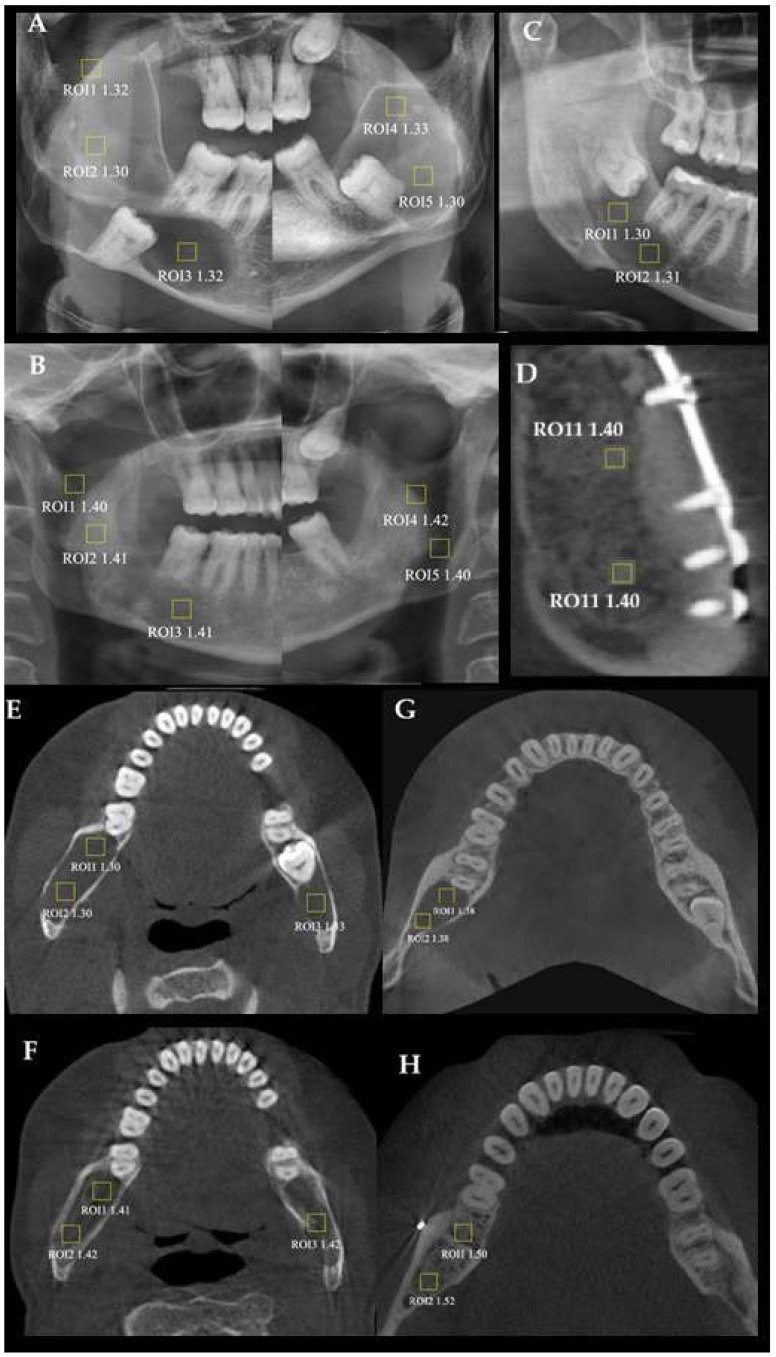
FA assessment of trabecular bone changes. (**A**,**B**,**E**,**F**) Case 1 and (**C**,**D**,**G**,**H**) Case 2 preoperative and 1-year postoperative panoramic and axial CT images at corresponding levels. Regions of interest (30 × 30 pixels) were analyzed using the box-counting method in ImageJ to evaluate longitudinal changes in FD, reflecting bone remodeling. A descriptive summary of lesion size, imaging follow-up intervals, cortical bone recovery, and fractal remodeling patterns in both cases is presented in Table 1.

**Table 1:** Imaging-based summary of follow-up and bone remodeling findings in the two cases.

Parameters	Case 1 (Two Lesions)	Case 2
**Initial Lesion Dimensions (cm)**	Lesion A: 3.8 × 2.0 × 3.8 Lesion B: 3.2 × 1.5 × 3.2	1.5 × 2.0 × 2.0
**Follow-up Time Points**	Preoperative, 6 months, and 1-year	Preoperative, 6 months, and 1-year
**Imaging Modalities Used**	OPG and CBCT	OPG and CBCT
**Cortical Bone Recovery**	Restored (confirmed via 1-year CBCT)	Restored (confirmed via 1-year CBCT)
**Bone Remodeling Pattern**	Gradual FD Progression	Sharper Postoperative FD Rise
**Fractal Analysis Status**	Quantitatively Confirmed	Quantitatively Confirmed

OPG, Orthopantomogram; CBCT, Cone-Beam Computed Tomography; FD, Fractal Dimension. Initial dimensions were measured at the widest clinical and radiological points.

Both cases emphasized the importance of CBCT-based assessment of bone loss, lesion extent, and proximity to vital structures in surgical planning. In the staged marsupialization approach, gradual decompression promoted bone self-healing and created a safer environment for subsequent definitive treatment, although this strategy required prolonged follow-up and did not eliminate the risk of complications such as pathological fracture. In contrast, one-stage cystectomy with ostectomy, bone grafting, and patient-specific mesh plating provided immediate structural support and a stable reconstructive outcome, but at the cost of greater surgical intensity and the possible need for later plate removal. Despite these differences, both management pathways shared the same therapeutic objective: preservation or restoration of mandibular continuity, functional stability, and adequate bone architecture to support long-term occlusion and mastication.

## 3. Discussion

Odontogenic cysts account for a substantial proportion of jaw lesions and are encountered more often than odontogenic tumors [13]. The presence of secondary infection, cortical bone thinning with expansion, and root resorption may complicate the radiologic distinction between odontogenic cysts and benign odontogenic tumors [14]. Among odontogenic cysts, periapical (radicular) cysts are reported as the most prevalent, followed by dentigerous cysts, which most commonly involve mandibular third molars. From another perspective, studies focusing specifically on impacted third molars consistently identify odontogenic cysts as the predominant associated pathologic entities, with dentigerous cysts representing the most frequent diagnosis [13,14,15].

One lesion in Case 1 merits separate comment because a glandular odontogenic cyst is both uncommon and diagnostically deceptive. Recent literature emphasizes that GOC often shows nonspecific radiolucent features with substantial overlap with odontogenic keratocyst, ameloblastoma, and other cystic jaw lesions, especially when lesions are large or multilocular and accompanied by cortical expansion, tooth displacement, or root resorption [16,17,18,19]. At the same time, recurrence remains a genuine concern after conservative management. A 2024 systematic review [20] of recurrent GOCs and a 2025 ten-case series [21] both stressed that preoperative imaging alone is insufficient for reliable diagnosis and that long-term follow-up is warranted even after apparently adequate treatment. In the present report, the contralateral GOC therefore reinforces the central message that imaging can guide suspicion and operative planning, but histopathologic confirmation remains decisive.

In the present cases, postoperative radiologic follow-up suggested different patterns of defect filling according to the selected treatment pathway: marsupialization/decompression was associated with predominantly peripheral-to-central trabecular infill, whereas primary enucleation or graft-assisted reconstruction showed a different radiologic course, with earlier central obliteration or more homogeneous radiopacity [22]. These observations are descriptive and should be interpreted in light of lesion-specific biology, baseline defect morphology, and the reconstructive method used. The literature also supports marsupialization/decompression as a useful bone-preserving strategy in selected large lesions rather than as a universal definitive treatment. Consolo et al. [7] reported a mean volume reduction of 63.9% after marsupialization, with greater reduction associated with longer treatment duration and larger baseline volume. Bellini et al. [22] later suggested that, for large mandibular cysts, around 8 months may be a practical interval for definitive enucleation, with time to CBCT follow-up being the main factor associated with volume reduction. Dereci et al. [23] similarly reported that both decompression and enucleation reduced cystic defects, without superiority of one method over the other at 12 months.

Our cases illustrate how subtle radiologic parameters, particularly inferior border deformation, lingual cortical discontinuity, and three-dimensional nerve displacement, were decisive in selecting between staged decompression, primary enucleation, or immediate radical surgery with prophylactic fixation. However, these radiologic parameters were interpreted together with pathological diagnosis, particularly in the OKC case, where biopsy confirmation supported peripheral ostectomy because of the lesion’s recurrence potential. Beyond illustrating rare cyst presentations, the present cases provide an imaging-oriented framework for interpreting postoperative bone regeneration after different surgical intensities, emphasizing how CBCT-based assessment can guide selection between prolonged decompression protocols and one-stage radical resection with grafting and PMP.

In the surgical management of mandibular odontogenic cysts, basing the therapeutic approach solely on lesion size is inadequate; rather, the critical determinants are the amount of residual cortical bone and the overall biomechanical integrity of the mandible following surgery. The literature demonstrates that, in carefully selected cases where cortical continuity is preserved and the inferior mandibular border remains stable, enucleation alone may constitute a sufficient treatment modality. Previous reports indicate that large cystic lesions may be managed without prophylactic plating in selected cases [16], whereas in situations with marked cortical thinning and increased fracture risk, prophylactic stabilization with a load-bearing titanium plate prior to enucleation has also been recommended [17]. Considered together, the extent of cortical bone loss may suggest which type of approach would be more appropriate. Radiologic “red flags” include inferior border thinning or bowing, cortical perforation, mandibular canal engulfment, impacted tooth presence, ramus height involvement, and bilaterality on CBCT [18,19,20]. These descriptors are presented as practical imaging-based observations derived from the current cases and the literature, not as validated universal thresholds or a formal treatment algorithm.

In the grafted area, increased radiopacity on follow-up CBCT was interpreted cautiously. Radiologic signs suggesting bone healing included progressive defect fill, continuity of the radiopaque area with the surrounding native trabecular bone, cortical reconstitution, and reduction of the original cystic cavity. However, residual or partially remodeled graft particles may also contribute to increased radiopacity, particularly when they appear as discrete granular or hyperdense foci rather than as continuous trabecular bone. Therefore, the grafted site should be interpreted as radiologic consolidation and graft incorporation/remodeling rather than histologically proven complete new bone regeneration. FA should therefore be regarded as an exploratory supportive method rather than a definitive biological endpoint. In this case-based observation, it was used to complement, but not replace, visual CBCT assessment and clinical follow-up.

Both the allogeneic bone used and the patients’ self-healing bone cavity healing and their outcomes can be easily assessed in radiological appearance on CBCT imaging, as well as in the estimation of bone remodeling. Another important issue that requires further study is bone integrity and differences in radiological healing assessed on CBCT based on allogeneic, xenograft, and autograft bones used in selected mandibular defects [23,24,25].

It is important to acknowledge that the biological heterogeneity of the lesions in this report, including dentigerous, glandular odontogenic, and odontogenic keratocysts, introduces confounding variables. Each lesion type possesses inherent differences in aggressiveness and recurrence potential, which may independently influence the observed bone healing signatures. While this case report focuses on the mechanical and radiological aspects of bone regeneration, these biological factors should be considered when interpreting the results. Because surgical treatment planning in large odontogenic cystic lesions is initiated primarily according to clinical presentation, radiologic extent, cortical bone status, and relationship to vital structures before definitive histopathologic confirmation is available, the present comparison is intended to reflect differences in surgical decision-making pathways rather than a pathology-matched comparison of biologically identical lesions. Accordingly, the postoperative radiologic patterns observed in these cases should not be interpreted as consequences of surgical strategy alone, but rather as the result of an interaction between lesion-specific biology, baseline structural conditions, and case-specific surgical management.

A further limitation is that the present report was not designed as a long-term recurrence or survival study. Although early radiologic healing and structural stability could be documented during the available follow-up period, a 1-year follow-up is insufficient to draw conclusions regarding long-term recurrence risk, particularly for lesions with recognized recurrence potential such as OKC and GOC. Therefore, the absence of recurrence during the reported follow-up should be interpreted only as an early clinical and radiologic finding, and prolonged surveillance remains necessary. Because this is a case report, FA was used only as a supportive descriptive tool rather than as an inferential research outcome. The results should therefore be interpreted cautiously, as FA values may be influenced by ROI selection, radiographic quality, projection-related factors, fixation materials, and local anatomical structures. Conventional radiologic follow-up with panoramic radiography and CBCT remains the primary basis for evaluating postoperative bone condition in the present cases.

These cases highlight that CBCT-based assessment of lesion proximity to vital structures is critical in guiding surgical decision-making. Treatment strategies may range from marsupialization to primary enucleation, with additional stabilization procedures such as intermaxillary fixation considered when structural integrity is compromised. Despite advanced imaging, histopathologic evaluation remains essential for definitive diagnosis. Moreover, differences in bone regeneration and density between grafted and non-grafted approaches underscore the importance of individualized treatment planning and radiologic follow-up. However, safety outcomes should be interpreted cautiously, as the present cases did not share identical postoperative morbidity profiles: Case 1 showed no sensory disturbance, whereas Case 2 involved postoperative neurosensory deficit with full recovery of inferior alveolar nerve function but only partial recovery of lingual nerve function. Accordingly, several key lessons can be derived from these cases.

- CBCT-Guided Follow-up: High-resolution assessment of cortical integrity and lesion proximity to vital structures is paramount in selecting between staged decompression, primary enucleation, or radical resection [4].
- Biomechanical Integrity: Treatment strategies must prioritize the residual bone volume; while marsupialization preserves bone and nerves, radical approaches with PMP are necessary when structural stability is compromised [9].
- Diagnostic Priority: Despite the utility of quantitative imaging, histopathologic evaluation remains the definitive gold standard for diagnosis and final treatment planning [20].
- Quantitative Monitoring: The integration of FA provides an objective mathematical framework to monitor bone regeneration patterns, offering a higher level of precision than qualitative radiological assessment alone.

## 4. Conclusions

Regular radiographic monitoring of impacted teeth, even in the absence of symptoms, is essential for early detection of associated pathologies. In the present cases, management strategies ranging from marsupialization with enucleation to one-stage radical resection with PMP were associated with favorable bone healing when tailored to the specific biomechanical and radiological features of each lesion; however, postoperative morbidity, particularly neurosensory outcome, differed between cases. Early diagnosis and appropriate timing of intervention were key factors in achieving favorable outcomes. CBCT may provide a more detailed postoperative evaluation of bone healing, allowing detailed assessment of trabecular reorganization and bone regeneration, which cannot be adequately appreciated on panoramic radiographs. Furthermore, treatment selection should not rely solely on radiologic findings such as bone integrity and fracture risk, but should also incorporate individualized considerations through surgeon–patient discussion.

## Figures and Tables

**Figure 1:**
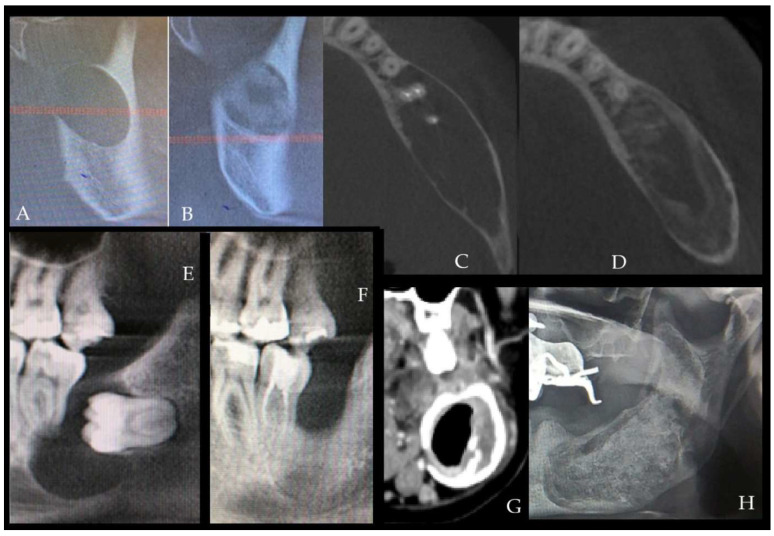
Spectrum of clinical and surgical scenarios influencing bone healing in mandibular cystic and tumor-like lesions. Bone healing after mandibular surgery depends not only on the histopathologic nature of the lesion but also on the extent of surgery, presence of infection, residual cortical support, lesion size and shape, and the reconstructive strategy selected for each case. The illustrative examples presented in (**A**–**H**) summarize different healing patterns and treatment philosophies that may be encountered in mandibular surgery. ((**A**) Preoperatively, (**B**) Postoperatively) A closed odontogenic keratocyst (OKC) of the mandibular ramus with preserved overall contour, treated by cystectomy and peripheral burr ostectomy, with the cavity tightly closed and left for spontaneous healing based on blood clot formation (red lines are the horizontal orientation lines in CBCT). ((**C**) Preoperatively, (**D**) Postoperatively) A solitary bone cyst (SBC) treated by curettage and stimulation of bleeding, followed by satisfactory spontaneous bone regeneration without the use of grafting materials. ((**E**) Preoperatively, (**F**) Postoperatively) A follicular cyst associated with an impacted molar, in which the bone cavity was left empty because of purulent content, yet subsequent follow-up demonstrated satisfactory bone healing without additional reconstructive materials. ((**G**) Preoperatively, (**H**) Postoperatively) A large OKC involving the left mandibular body and ramus was managed in a staged manner with initial biopsy and marsupialization, followed by delayed revision surgery, peripheral ostectomy, bone debridement, and placement of allogeneic bone graft, resulting in favorable healing. These representative scenarios demonstrate that mandibular bone healing may be achieved through different pathways, ranging from spontaneous secondary healing to graft-assisted reconstruction. The final decision is influenced by local bone conditions, including cortical preservation or loss, integrity of the inferior mandibular border, risk of pathological or iatrogenic fracture, lesion extension beyond bone boundaries, proximity to the inferior alveolar nerve and adjacent teeth, the presence of infection or soft-tissue involvement, and suspected or confirmed pathological diagnosis when available.

**Figure 2:**
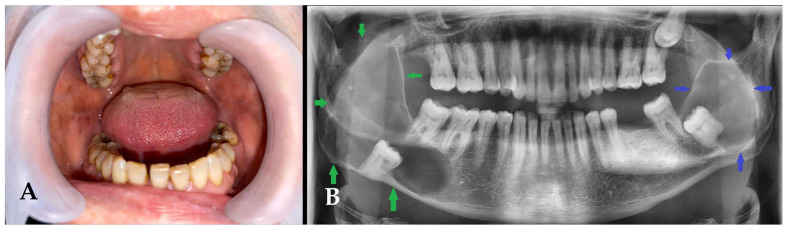
(**A**) Initial intraoperative photograph, (**B**) Initial scan—orthopantomogram (OPG).

**Figure 3:**
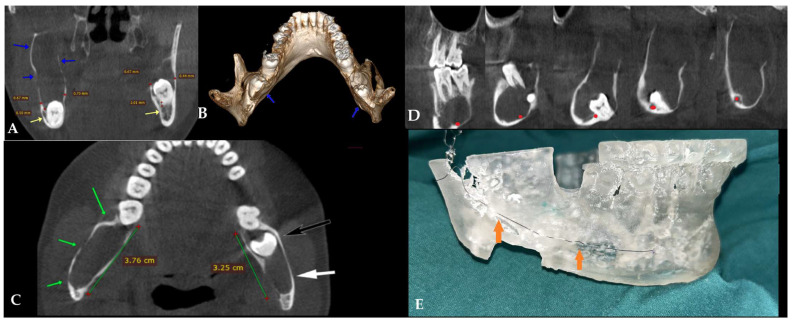
(**A**) Coronal CBCT sections show mediolateral evaluation of the cystic lesion and adjacent cortical plates. Blue arrows indicate the measured preoperative buccal and lingual cortical bone thickness in the mandibular ramus region: right buccal cortex, 0.67 mm; right lingual cortex, 0.70 mm; left buccal cortex, 0.44 mm; left lingual cortex, 0.67 mm. Yellow arrows demonstrate the close proximity of the IAN to the teeth associated with the cystic lesions, with the shortest measurable lesion/tooth complex–IAN distance of 0.50 mm on the right and 2.01 mm on the left. CBCT images (**B**) show thinning of the lingual cortical plate bilaterally (arrows) and impacted third molars (#48 and #38). Axial CBCT image (**C**) demonstrates thinning of the buccal cortical bone (arrows) and the measured dimensions of the lesion. Sagittal CBCT views (**D**) show red dots representing the IAN. The patient-specific transparent 3D model (Biotechnica Engineering Co., Ltd., Turkiye) shows the wire (**E**) and the inferior alveolar nerve (indicated by orange arrows).

**Figure 4:**
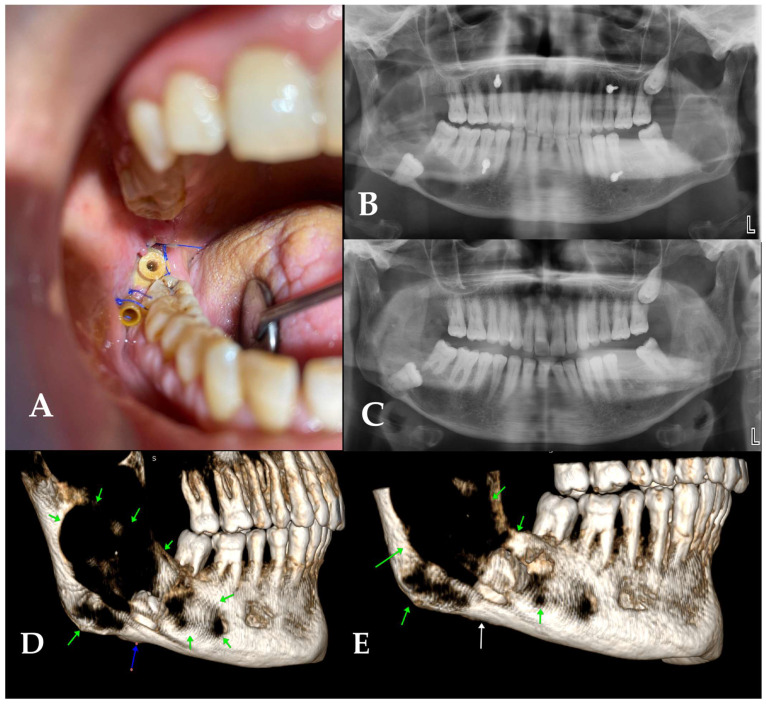
Clinical and radiographic course of the marsupialization phase in Case 1. (**A**) Clinical view of the right mandibular marsupialization site after placement of two drains. (**B**) Immediate postoperative panoramic radiograph after left-sided enucleation/extraction and right-sided marsupialization, showing the drain in situ; the absence of tooth #38 confirms that this image represents a postoperative/control stage rather than the initial preoperative condition. (**C**) Six-month control panoramic radiograph demonstrating interval healing. (**D**) Pre-marsupialization vertical position of tooth #48 (blue arrow) and boundaries of the right-sided cystic lesion (green arrows). (**E**) Six months after marsupialization, radiographic evaluation shows reduction in cystic dimensions (green arrows) and vertical eruption of tooth #48 (white arrow), facilitating safer definitive surgical management.

**Figure 5:**
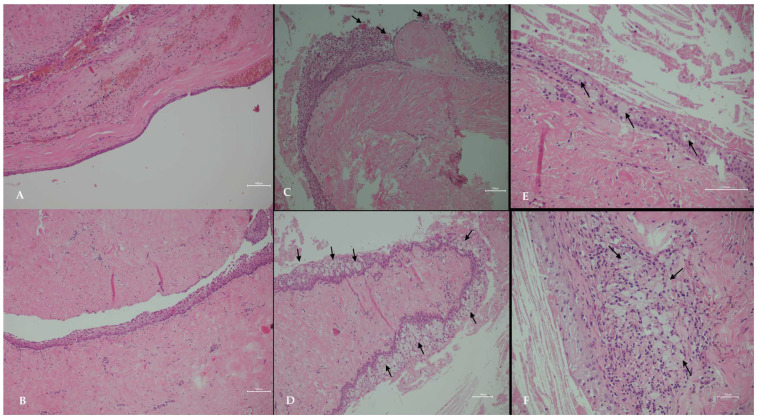
Histopathological features of bilateral mandibular cystic lesions. (**A**,**B**) Right-sided cyst specimen. (**C**–**F**) Left-sided cyst specimen showing characteristic features of a glandular odontogenic cyst with intraepithelial microcysts and mucous goblet cells (black arrows) (H&E, ×100–×200).

**Figure 6:**
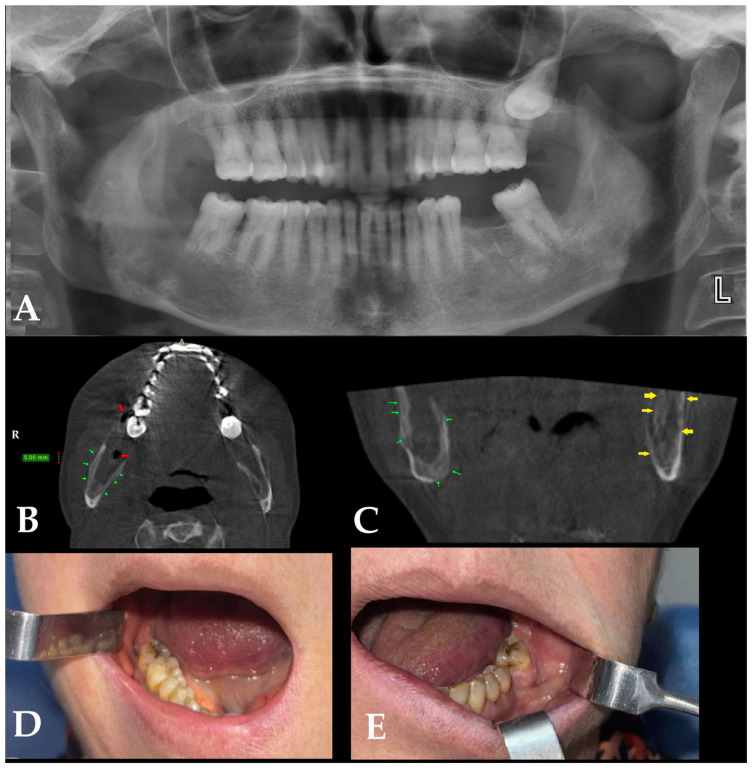
OPGs taken 1 year after surgery (**A**). New bone formation on CBCTs 1 year after surgery (**B**,**C**). No postoperative infection, recurrence, or neurological complications were observed (**D**,**E**).

**Figure 7:**
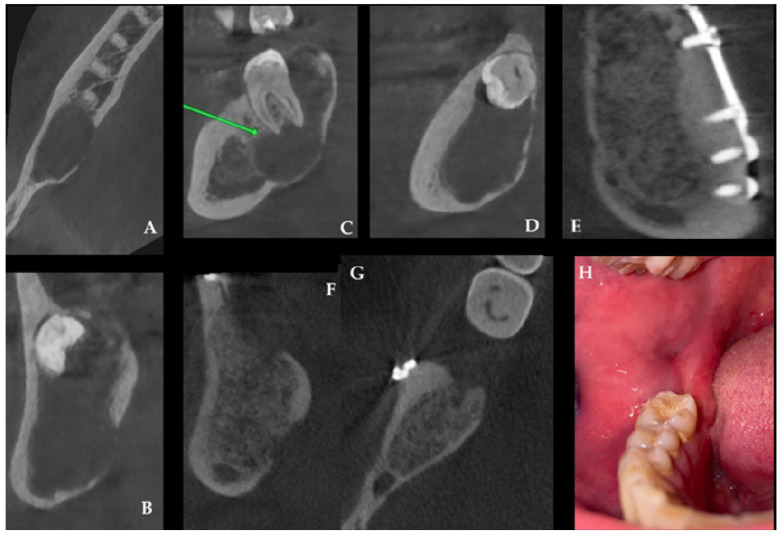
One-stage radical management of a mandibular OKC. (**A**–**D**) Preoperative clinical and CBCT findings demonstrating a large osteolytic lesion in the right mandibular body/angle region with lingual cortical loss and displacement of the inferior alveolar nerve. (**E**) Immediate stabilization with a 2.0 rigid preventive mandibular plate and reconstruction using a fresh-frozen allogeneic bone graft mixed with autologous blood. (**F**–**H**) Postoperative and one-year follow-up demonstrated uneventful healing, radiologic consolidation of the grafted defect, and no evidence of recurrence. Green arrow points on a small osseous vessel adjacent to the surgical site.

## Data Availability

The datasets used and/or analysed during the current study are available from the corresponding author upon reasonable request due to privacy considerations. (The following concerns the access to full CBCT/radiology study for comparison).
